# Semantic memory: nouns and action verbs in cognitively unimpaired individuals and frontotemporal lobar degeneration

**DOI:** 10.1590/S1980-57642013DN70100008

**Published:** 2013

**Authors:** Leticia Lessa Mansur, Maria Teresa Carthery-Goulart, Valéria Santoro Bahia, Thomas H. Bak, Ricardo Nitrini

**Affiliations:** 1 PhD. Department of Physiotherapy, Speech Therapy and Occupational Therapy, University of São Paulo, São Paulo SP, Brazil.; 2 PhD, Behavioral and Cognitive Neurology Unit, Department of Neurology, Hospital das Clínicas, University of São Paulo School of Medicine, São Paulo SP, Brazil. Cognition and Complex Systems Unit and Center for Mathematics, Computing and Cognition, Federal University of ABC (UFABC), Brazil.; 3 MD, PhD. Behavioral and Cognitive Neurology Unit, Department of Neurology, Hospital das Clínicas, University of São Paulo School of Medicine, São Paulo SP, Brazil. Pontifícia Universidade Católica de São Paulo- PUC-SP, São Paulo SP, Brazil.; 4 University of Edinburgh School of Psychology, Philosophy & Language Sciences and Centre for Clinical Brain Sciences, Edinburgh UK.; 5 Full Professor. Department of Neurology. School of Medicine. University of São Paulo, São Paulo SP, Brazil.

**Keywords:** cognition, language, memory, neuropsychological tests, age, schooling

## Abstract

Among the instruments to evaluate semantic memory, the Pyramids and Palm Trees (PPT) and the Kissing and Dancing (KDT) tests are widely used but none have a performance referential for cognitively normal and impaired Brazilian populations.

**OBJECTIVE:**

[A] To study these two tests in a sample of young healthy Brazilian individuals living in São Paulo; [B] To apply the results to the evaluation of two cases diagnosed with frontotemporal lobar degeneration.

**METHODS:**

We evaluated 50 normal participants (41 females and 9 males) aged between 20-63 years, with schooling level of 14-20 years. In addition, two individuals diagnosed with frontotemporal lobar degeneration were examined, one with behavioral-variant frontotemporal dementia and the other with semantic dementia.

**RESULTS:**

On the two tests, no effects of age, gender and schooling on the performance of normal individuals were observed. According to the performance of the sample of controls, scores below 46 points on the PPT and below 47 on the KDT are suggestive of deficits in semantic memory. The analyses of both cases indicated double dissociation in establishing associations between nouns and action verbs. Although the two patients had low scores on both tests, the patient with behavioral-variant frontotemporal dementia performed better on the PPT compared to the KDT, while the patient with semantic dementia showed the reverse, performing better on the KDT.

**CONCLUSION:**

The PPT and KDT are suitable tests for use in the Brazilian population, with minimal need for adjustments. They are applicable tools both for cognitive assessment and research in semantic memory. In the present study, we obtained representative values of performance for cognitively unimpaired individuals and demonstrated the utility of these instruments for cognitive assessment of patients with FTLD.

## Abstract (pt)

Entre os instrumentos dirigidos à avaliação da memória semântica, temos os testes Pirâmides e Palmeiras (*Pyramids and Palm Trees* - PPT) e o Beijando e Dançando (*Kissing and Dancing* - KDT), para os quais não existem referências de desempenho de população brasileira normal e patológica.

**OBJETIVO:**

[A] Estudar esses dois testes em amostra de indivíduos jovens, sadios brasileiros, residentes na cidade de São Paulo; [B] Aplicar os resultados na avaliação de dois casos diagnosticados no espectro da degeneração lobar frontotemporal.

**MÉTODOS:**

Avaliamos 50 participantes normais (41 mulheres e 9 homens) com idade entre 20-63 anos e escolaridade entre 14-20 anos. Adicionalmente foram examinados dois sujeitos com diagnóstico de degeneração lobar frontotemporal: um com demência frontotemporal-variante comportamental e outro com demência semântica.

**RESULTADOS:**

Não observamos efeitos de idade, gênero e escolaridade na performance dos indivíduos normais nos dois testes. Obtivemos os valores de 46 pontos no PPT e 47 no KDT como indicativos de risco de déficit em memória semântica. A análise dos dois casos mostrou dupla dissociação na determinação de associações entre substantivos e verbos. Embora os pacientes apresentassem escores rebaixados nos dois testes, o paciente com demência frontotemporal-variante comportamental obteve melhor desempenho no PPT quando comparado ao KDT e o paciente com demência semântica mostrou padrão inverso, com melhor desempenho no KDT.

**CONCLUSÃO:**

Os testes PPT e KDT são aplicáveis à população brasileira, com necessidade de mínimos ajustes. Foi possível obter valores representativos de desempenho de população sadia, assim como vislumbrar o emprego em avaliações cognitivas de memória semântica nas DLFT.

## INTRODUCTION

Concepts are a means of categorizing the world in order to better understand it and establish communication references.^1^ Concepts can be represented by words and modern views of this knowledge allow for network organizations, such that strong connections are present among the components of a semantic representation distributed by one word.^2^

Semantic memory has been widely investigated in brain lesions and through models derived from computational theory.^1^ In the context of Neuropsychology, we seek to diagnose the integrity of representations or dissociations present in different clinical profiles resulting from vascular, traumatic or degenerative lesions. Two instruments used for this purpose include the Pyramids and Palm Trees (PPT)^3^ and the Kissing and Dancing (KDT) tests.^4^

The PPT is used to evaluate the ability to access semantic representations. The test includes stimuli - nouns - which can be represented by drawings; and for this reason it does not allow conclusions to be drawn about lexical comprehension of abstract concepts. The test involves several types of knowledge and associations, as well as temporary retention of the correct semantic information from three items displayed on each card. Individuals are required to select relevant information to establish the association between two out of the three items. Oral expression is not needed, which makes the test suitable and easier to apply in patients with difficulties in oral language production. The KDT follows the same principles. However, in this test action verbs are the concepts represented.

Dissociation in the processing of nouns and verbs was first recognized in naming tasks performed by aphasic patients^5,6^ and confirmed by studies in patients with motor neuron diseases^7^ and frontotemporal dementia.^4,8-11^ Recent imaging studies have demonstrated the neural bases of this dissociation.^12-14^

The evaluation of noun and verb representations have also contributed to the establishing of anatomoclinical correlations and to the follow-up of patients with neurological lesions.^10,15-17^

The PPT was originally standardized for use in the Quebec-French^18^ and Spanish-speaking populations,^19^ as well as in a population of elderly Italians.^20^ These studies recognized the effects of age, gender and schooling on performance, justifying cultural adaptations. To our knowledge, no norms for the performance of healthy Brazilian individuals on the PPT and KDT have been published. It is important to determine these references in order to interpret the results attained by patients, as cultural differences can interfere with performance. In this context, our objectives were: [A] to analyze performance on the PPT and KDT of a group of Brazilian individuals without cognitive deficits living in the city of São Paulo; [B] to compare the performance of this group with the results of two patients diagnosed with two different frontotemporal lobar degeneration syndromes, namely, semantic dementia (SD) and behavioral-variant frontotemporal dementia (bvFTD).

## METHODS

We assessed 50 healthy participants, 41 females and 9 males, whose socio-demographic characteristics are given in Table 1.

**Table 1:** Sociodemographic data of cognitively unimpaired individuals.

	**Mean**	**Standard Deviation**	**Range**
Age	26.26	9.40	20-63
Education	15.78	2.66	14-21
**Gender**	**N**	**%**	
Male	9	17.6	
Female	41	82.4	

Additionally, two patients were evaluated, one diagnosed with bvFTD and the other with SD.

**Instruments.** The PPT and KDT tests contain 52 stimuli. Each display board contains black-and-white triad drawings of nouns (PPT) and verbs (KDT). On the upper half, a drawing of an item is presented; on the lower half there are two choices: the target and a distractor also represented by drawings. The item in the upper half needs to be analyzed in relation to the other two. The two choices consist of semantically coordinated items, while the item in the upper half belongs to a different semantic category. The correct choice is based on a semantic property or association shared by the target item and the item in the upper half.

Cognitively unimpaired individuals (controls) were tested collectively using the Microsoft Power Point program (97-2003) to project the stimuli for a period of 5 seconds on an enlarged screen. The subjects received a sheet, with the written name of the target-item plus the two options and were instructed to register their choice.

The patients were evaluated individually in a silent room using a conventional triad presentation on A6 boards. The following instruction was given: *"Here we have three drawings. You need to decide which of these on the bottom match the one on the top. Is it this one or the other"?* One point was given for each correct answer.

**Statistics.** Non-parametric tests were used in all comparisons. The Wilcoxon and Friedman tests were used to compare the performance between PPT and KDT, respectively, and to analyze item difficulty in both tests. The Mann-Whitney test was used to compare performances of men and women. Spearman's test was used to check for correlations among test performance, age and education.

This study is part of a larger research project on semantic memory approved by the Research Ethics Committee of the University of São Paulo City (Unicid) under nº CAAE 4461.0.000.107-10.

Patient data are given in descriptive form. Subjects were selected from a group of patients with dementia seen at the Cognitive Neurology Unit, Neurology Service, Hospital das Clínicas of the University of São Paulo School of Medicine.

## RESULTS

**Controls.** The results on the PPT and KDT for the sample of cognitively unimpaired individuals are shown in Table 2. Participants achieved better scores on the KDT (48.31) compared to the PPT (47.62) (p=0.009).

**Table 2:** Performance of cognitively unimpaired individuals.

N=50 subjects	Mean	Std. Deviation	Range	Percentiles		
				**25^th^**	**50^th^ (Median)**	**75^th^**
Pyramids and Palm Tree Test	47.62	2.55	38-52	46.00	48.00	49.25
Kissing and Dancing Test	48.31	3.25	37-52	47.00	49.00	50.25

There was no significant difference in performance between men and women on the PPT (p=0.096) or the KDT (p=0.725). In addition, Spearman correlations were not significant for the PPT and age (p=0.089), the PPT and education (p=0.096), the KDT and age (p=0.256), or the KDT and education (0.103).

It was possible to estimate the risk scores at the 25^th^ percentile: below 46 for the PPT and below 47 for the KDT.

There was an item difficulty effect on the PPT (p< 0.01) and KDT (p<0.01). Qualitative analysis revealed that the entire sample scored correctly on only 5 items of the PPT (items 1, 10, 35, 36, 51) and 3 items of the KDT (items 45, 50 and 51). Table 3 shows those items which caused significantly more errors in the sample.

**Table 3:** Qualitative analysis of correct answers by item.

**PPT items**		**Incorrect choices**	**% of sample**	**p**
4	Thimble	10	19.6	p<0.01
8	Mice	6	11.8	p<0.05
12	Pyramid	10	19.6	p<0.01
15	Web	7	13.7	p<0.05
16	Windmill	20	39.2	p<0.01
17	Carrot	13	25.5	p<0.01
20	Ring	7	13.7	p<0.05
30	Eggs	9	17.6	p<0.01
31	Puddle	6	11.8	p<0.05
40	Acorns	29	56.8	p<0.01
41	Baby	6	11.8	p<0.05
46	Padlock	6	11.8	p<0.05
48	Bellows	12	23.5	p<0.01
49	Mask	14	27.5	p<0.01
52	Eskimo	16	31.4	p<0.01
**KDT items**		**Incorrect choices **	**% of sample**	**p**
1	Writing	7	13.7	p<0.05
2	Washing	11	21.6	p<0.01
3	Kissing	7	13.7	p<0.05
5	Shaving	8	15.7	p<0.01
6	Reading	7	13.7	p<0.05
7	Eating	7	13.7	p<0.05
8	Painting	8	15.7	p<0.01
13	Smiling	8	15.7	p<0.01
15	Shutting	11	21.6	p<0.01
16	Pushing	10	19.6	p<0.01
23	Skiing	8	15.7	p<0.01
31	Dusting	6	11.8	p<0.05
48	Watering	9	17.6	p<0.01

**Case study 1.** This case involved a 54-year-old, right-handed female patient, a Brazilian Portuguese speaker, with 11 years of schooling, diagnosed with bvFTD 2 years earlier.

The family reported that in June 2009 the patient exacerbated some traits of personality, becoming more impatient. Changes of behavior were noted, with disinhibition, loss of pudency and common sense, exposing herself to danger, and excessive spending. In addition, the family reported that the patient had become repetitive, lost the ability to conduct dialogue and became dependent and without initiative, even for basic activities.

Two years after symptoms onset (August, 2009) the patient underwent neurological examination. On the Mini-Mental State Examination she obtained a score of 26/30 and was classified as mild-moderate from a functional standpoint, according to CDR-L^21^ criteria.

**Figure 1:**
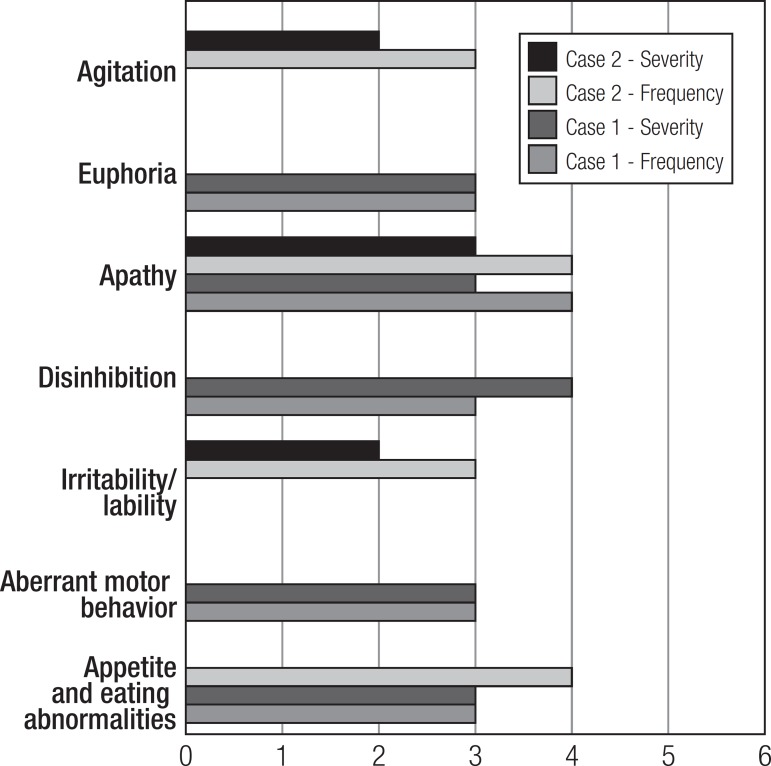
Neuropsychiatric inventory: frequency and severity of symptom* domain. *Symptoms not reported: night-time behavior disturbances, anxiety, dysphoria, hallucinations, and delusions.

**Figure 2:**
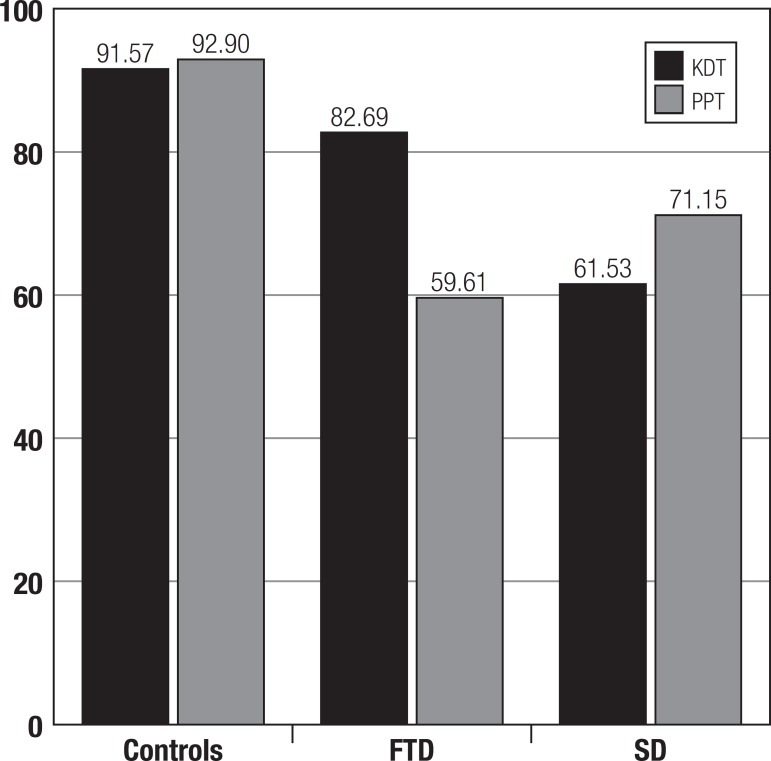
Performance (%) of controls and patients on PPT and KDT.

The behavioral (Neuropsychiatric Inventory - NPI),^22^ functional (Disability Assessment in Dementia - DAD)^23^ and cognitive (Addenbrooke Cognitive Examination Revised - ACE-R)^24^ assessments indicated impairments in these three aspects (Tables 4, 5 and Figures 1, 2).

**Table 4:** Addenbrooke Cognitive Examination - Revised: performance of bvFTLD and SD patients.

	Orientation	Memory	Fluency	Language	Visuospatial
Cut-off*	17	15	8	22	13
bvFTD (Case 1)	16	14	6	17	16
SD (Case 2)	9	3	0	10	12

* Reference value of Brazilian cognitively unimpaired individuals.^22^

**Table 5:** Disability Assessment of Dementia (DAD)23: performance of bvFTLD and SD patients.

	Activities	Max score	bvFTLD Case 1	SD Case 2
**Basic **	Hygiene	7	7	7
	Dressing/undressing	5	5	5
	Continence	2	2	2
	Eating	3	2	2
**Instrumental **	Meal preparation	3	2	3
	Telephoning	4	4	4
	Going on outings	5	3	5
	Finance and correspondence	4	3	0
	Medication	2	2	0
	Leisure and housework	5	2	2
	Total N/D		2	0
Total DAD score		**40**	**32**	**30**
			**84.21%**	**75%**

*Magnetic resonance imaging* - MRI disclosed atrophy of frontal and anterior temporal regions bilaterally, consistent with the diagnosis of bvFTD.

*Performance on PPT and KDT* - The patient exhibited greater impairment in verb processing than in noun processing. She correctly associated 43 (82.7%) stimuli (-1.81 standard deviation-SD compared to controls) on the PPT and 31 (59.6%) stimuli on the KDT -- 5.32 SD).

**Case study 2.** This case involved a 68-year-old, right-handed, female patient, with 11 years of schooling, diagnosed with SD 4 years earlier. Initially, her main difficulties were related to word-finding problems, remembering names of people and streets, associated with depressive symptoms. The patient was aware of her difficulties and became increasingly isolated and dependent for daily activities involving language and placing a high demand on executive functions. On the Mini-Mental State Examination, she scored 16/30 and was classified as mild-moderate from a functional standpoint, according to CDR-L criteria.

The patient exhibited cognitive impairment (Addenbrooke Cognitive Examination Revised - ACE-R) and functional difficulties (Disability Assessment in Dementia - DAD), as well as deviant behavior (Neuropsychiatric Inventory - NPI) (Tables 4, 5 and Figure 1).

*Magnetic resonance imaging* - MRI disclosed atrophy in the anterior temporal lobes, predominantly to the left side, consistent with the clinical diagnosis of SD.

*Performance on PPT and KDT* - The patient obtained a score of 32 (61.53%) on the PPT (-5.32 SD) and 37 (71.15%) on the KDT (-3.48 SD).

The performance of the two patients on the PPT and the KDT tests can be seen in Figure 2.

This Figure illustrates the performance of controls showing no marked differences in PPT and KDT scores, along with the contrasting results of the two patients, evidencing a double dissociation involving association of concepts related to actions (KDT) and nouns (PPT).

## DISCUSSION

The sample evaluated was homogeneous, comprising predominantly young female individuals with a high level of schooling, according to Brazilian standards. Our expectation was to obtain results that reflected performance without interference of age and schooling. This expectation was confirmed. The influence of gender noted in standardization for the Spanish language^18^ was not observed in our sample, although there was a predominance of females.

Schooling did not influence performance on the PPT or KDT. Our sample was highly homogenous with educational level of over 8 years. In a previous study, we found no schooling effect for the Brazilian population with over 8 years of schooling on a comprehensive language test.^25^ The Spanish study found a correlation between performance and years of schooling; however, there was an interaction of this effect with age, as the oldest had less schooling.^19^ In the Italian study, effects of both age and schooling were observed.^20^

The scores obtained by the Brazilian population were slightly lower than the values obtained by the English, Italian and Spanish populations on the PPT and the English population on the KDT. In the English population, the mean percentage of correct responses was 98-99% for the PPT and no individuals committed more than three errors.^3^ On the KDT, the mean was 96.15% correct responses.^4^ It is necessary to re-apply the test in Brazilian individuals, using written presentation of the stimuli to study verbal semantic memory in more depth and to exclude the impact of the cultural characteristics of graphic representation that the nonverbal test contains. Furthermore, contrary to the studies cited, Brazilians had slightly higher scores on the KDT than the PPT. It is possible that this result is due to a sampling effect: in as far as there is a tendency for performance with maximum scores and minimum deviation to gain significance. Moreover, cultural factors related to graphical presentation might be a factor. On this point, the relevance of studies on cross-cultural adaptation of neuropsychological instruments should be emphasized.

Regarding errors, 15 items on the PPT and 8 items on the KDT were associated with a high frequency of errors in controls and must be interpreted cautiously when testing brain-damaged individuals (Table 3). Difficulties perceiving the visual representation cannot solely be held responsible for the errors, as some items had simple representations, such as the *acorn* (PPT), a drawing that led 56.8% of the participants to make wrong associations. The same occurred with *eskimo*, responsible for 31.4% of the errors and *mask*, responsible for 27.5% of the errors. For the three stimuli, the principal factor that seemed to induce the wrong answer was semantic knowledge and cultural experience held with respect to the stimuli and the possibility of association (*acorn* and *eskimo* are not part of the universal culture of most Brazilian participants). Another aspect to be considered as exerting a high impact is presentation time; this must have had an impact on performance, especially for those items considered "easy" (from a conceptual point of view), but whose visual complexity required greater analysis time.

Future studies need to be performed to explore these differences in a larger sample including individuals with lower educational levels and elderly subjects in a gender-balanced sample.

A pattern of double dissociation was noted in the bvFTD and SD patients. Although the patients presented worse results on both tests compared to controls, the impairment was more severe for a specific category in each case, either verbs or nouns. The bvFTD patient performed worse than expected on verb processing yet was close to the mean of controls on the PPT. Furthermore, her profile was qualitatively different from controls whose best performance was on the KDT. These results are similar to the findings of Bak and Hodges in an English population.^4^ The specific deficit in verb processing in patients with FTD has been reported in other studies.^8,10^

In the case of the SD patient, performance on the KDT was impaired but less severely than on the PPT. Although semantic loss may predict decline on both tests (since we are analyzing semantic properties), knowledge of verbs was relatively more preserved. It is important to point out that noun deficits cannot be explained by visual recognition difficulties, since both tests are represented visually.

In addition to its contribution in terms of the assessment and applicability of these clinical tests, the present study raised an issue highlighted in recent years: the differential processing of nouns and verbs. The dissociation found in our cases is intriguing but should be interpreted cautiously. This discussion is broad, complex and beyond the scope of the present study. Recent investigations have sought to identify different methods and specialized networks of processing. A recent systematic review whose aim was to comprehensively analyze the methodological controversies, concluded that the evidence indicates the grammatical categories alone do not explain the different processes. On the other hand, the findings are consistent with the existence of different partially separable networks: frontoparietal action related to verb knowledge and inferotemporal, related to object knowledge (both underscoring the pragmatic / semantic foundation) and left prefrontal, including, in particular, the inferior frontal gyrus, related to the reliability of distributional information (syntactic behavior and morpho-syntactic marking).^26^ Following on from the present work, future studies of syntagmatic languages like Portuguese could be conducted.

The values obtained as a reference for performance of the normal Brazilian population proved able to differentiate the profiles in SD and bvFTD. As patients obtained lower-than-expected scores, it is relevant to further investigate the causes of the difficulty by addressing the points proposed by the authors of the PPT: [A] access to semantic/ item concept information; and [B] association capacity focusing on the relevant property relative to the target item, but not the distractor; [C] inhibition of other irrelevant semantic information such as similarities between target and distracters.^3^
